# The effect of standoff distance and surface roughness on biofilm disruption using cavitation

**DOI:** 10.1371/journal.pone.0236428

**Published:** 2020-07-30

**Authors:** N. Vyas, R. L. Sammons, S. A. Kuehne, C. Johansson, V. Stenport, Q. X. Wang, A. D. Walmsley

**Affiliations:** 1 School of Dentistry, College of Medical and Dental Sciences, University of Birmingham, Birmingham, United Kingdom; 2 Department of Prosthetic Dentistry/Dental Materials Science, Institute of Odontology, Sahlgrenska Academy, University of Gothenburg, Gothenburg, Sweden; 3 School of Mathematics, College of Engineering and Physical Sciences, University of Birmingham, United Kingdom; University of Notre Dame, UNITED STATES

## Abstract

Effective biofilm removal from surfaces in the mouth is a clinical challenge. Cavitation bubbles generated around a dental ultrasonic scaler are being investigated as a method to remove biofilms effectively. It is not known how parameters such as surface roughness and instrument distance from biofilm affect the removal. We grew *Strepotococcus sanguinis* biofilms on coverslips and titanium discs with varying surface roughness (between 0.02–3.15 μm). Experimental studies were carried out for the biofilm removal using high speed imaging and image analysis to calculate the area of biofilm removed at varying ultrasonic scaler standoff distances from the biofilm. We found that surface roughness up to 2 μm does not adversely affect biofilm removal but a surface roughness of 3 μm caused less biofilm removal. The standoff distance also has different effects depending on the surface roughness but overall a distance of 1 mm is just as effective as a distance of 0.5 mm. The results show significant biofilm removal due to an ultrasonic scaler tip operating for only 2s versus 15-60s in previous studies. The technique developed for high speed imaging and image analysis of biofilm removal can be used to investigate physical biofilm disruption from biomaterial surfaces in other fields.

## Introduction

More than 2 million dental implants are placed worldwide every year, with estimates suggesting an increase in the prevalence of dental implants in the USA of up to 23% in the next 10 years [1, 2]. Dental implants require maintenance over time to manage the formation of dental plaque [3]. Peri-implantitis is an inflammatory reaction with bone loss around the implant [4, 5]. If the plaque is not removed it could eventually lead to implant failure [6, 7] Dental plaque is a biofilm, a community of micro-organisms adhered to a surface [8]. Biofilms are more resistant to traditional antimicrobials than free floating bacteria, therefore physical methods of biofilm disruption are of interest to study [9–13].

There is no overall effective method to remove plaque biofilm from dental implants. Current clinical methods of removing dental plaque from implants include air polishing, the use of rotating titanium brushes or ultrasonic scalers with specialised tips to prevent scratching the implant surface [4, 14–18]. Ultrasonic scalers have a metal tip which vibrates at 25-50kHz [19]. Ultrasonic cavitation bubbles occur in the cooling water flowing over the tip of ultrasonic scalers [20, 21]. Cavitation bubbles are microbubbles that grow and collapse in a fluid when ultrasound is applied [22–24]. Biofilm disruption with cavitation can occur through various phenomena such as microstreaming and micro-jet impingement, leading to the production of shear forces which can weaken the attachment of biofilms to surfaces and eventually cause them to fragment and detach from the surface [25, 26]. Cavitation is used in other industries for ultrasonic cleaning [27–29], and could potentially be enhanced around ultrasonic scalers for use in cleaning biofilms from dental implants more effectively without damaging the surface.

**Fig 1:**
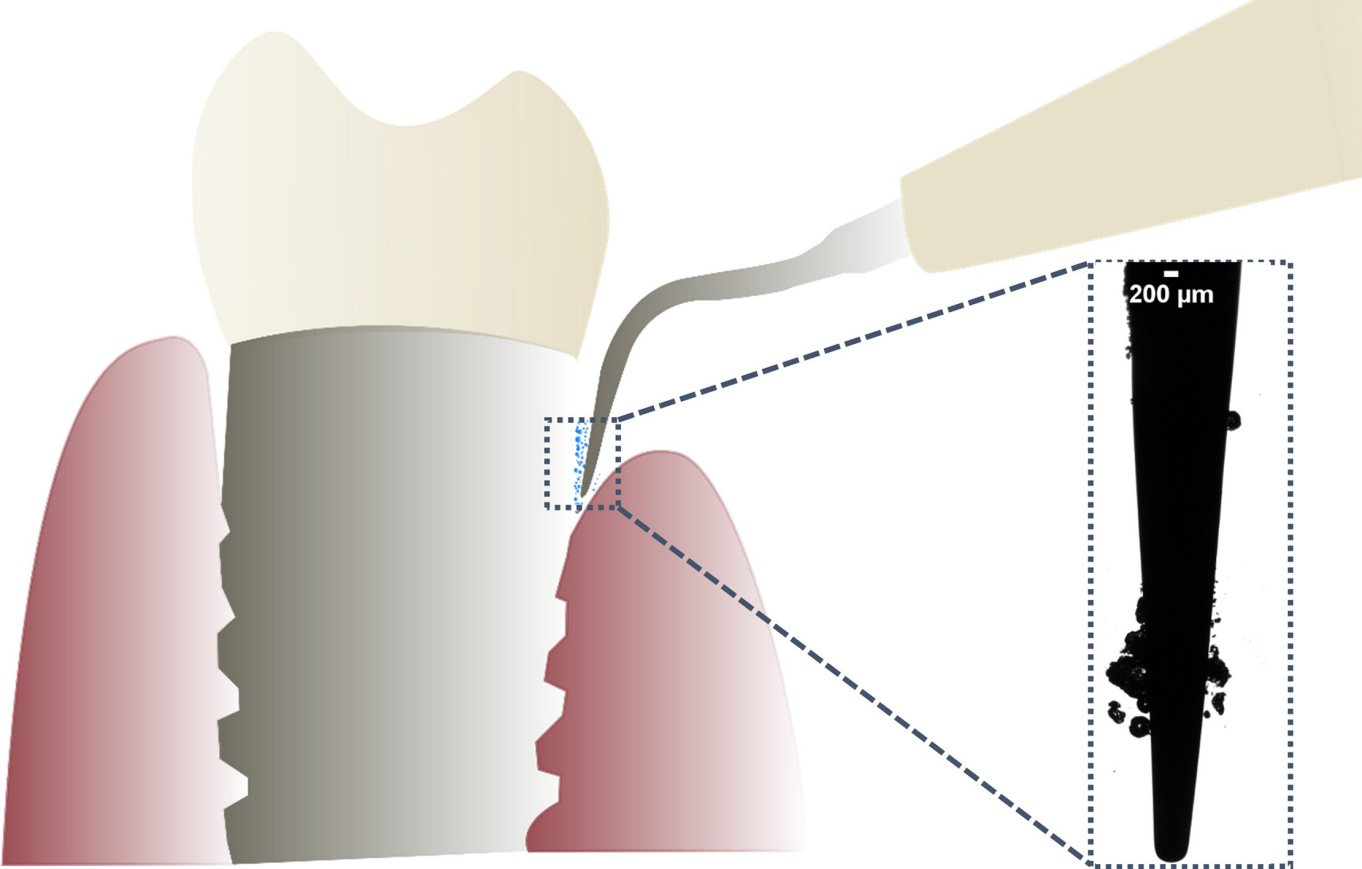
Schematic of how an ultrasonic scaler tip could be used to clean bacterial biofilm from dental implants in a non-touch mode using the cavitation generated around the tip. Inset: High speed camera image of cavitation bubbles occurring around the tip of the commercially available ultrasonic scaler tested in this study.

Ultrasonic cavitation around scalers has the potential to clean implants if the tip is used in a non-touch mode (Fig 1). To make this a clinically viable option, the effects of roughness *Ra* of biomaterial surfaces and the standoff distance *d* of a scaler tip from a biofilm require further understanding so the cavitation can be optimised accordingly. Previous research has shown that enhanced surface roughness increases bacterial attachment because of the larger contact area for bacterial attachment [30, 31]. Bacteria are also more protected from shear forces on a rough surface [30, 32]. In addition, the difficulty in cleaning the irregular profile of rough surfaces allows for biofilms to rapidly reform [32].

A high speed camera is able to image biofilm removal in real time over short time scales. This makes it possible to understand what causes bulk biofilm disruption and provides more information about the removal timescales. In addition it is possible to visualise the same area before and after cleaning. It can show how different biofilms behave in response to different removal techniques and is a valuable tool for understanding biofilm adhesion to surfaces and evaluating physical disruption methods. This information is critical to understanding how biofilm removal can be optimised. Since the use of a high speed camera and image analysis for measuring biofilm removal is a novel technique, to date there are a limited number of studies on the use of high speed imaging to quantitatively evaluate biofilm removal [33–35]. Such imaging and analysis protocols require development.

The aim of this study was to use high speed imaging and image analysis to record and evaluate the biofilm removal process from biomaterial surfaces of different roughness using cavitation from an ultrasonic scaler tip at different standoff distances. For this study, *Streptococcus sanguinis* biofilms were chosen over other species because this is a primary coloniser on dental implant surfaces. In addition it was chosen because compared to other streptococci species such as *Streptococcus mutan*s, which form clumps causing the biofilm to vary widely in thickness, *S*. *sanguinis* forms a relatively homogenous biofilm, and this would enable us to compare the effects of the surface roughness and tip distance more accurately. Only one species was considered for this study because multi species biofilms may have varying attachment forces at various locations in the biofilm, which may then cause false conclusions to be drawn about the effectiveness of cavitation on surfaces of different surface roughness.

## Materials and methods

### Preparation of the biomedical substrates

Two growth substrates were used in this study, and each had two variations of surface roughness. Biofilms were grown on transparent coverslips (13 mm diameter, Thermanox™, Nunc, ThermoFisher Scientific) and on Titanium (Ti) discs. The polymer coverslips are commonly used for *in vitro* biofilm research, therefore we included them in this study to show, if their material properties in relation to biofilm removal, are similar to Ti so they can be used in future *in vitro* experiments where a transparent substrate is required; for example high speed imaging at high shutter speeds, fluorescence imaging, or correlative studies using multiple imaging modalities. The Ti discs were used to mimic a dental implant surface.

The smooth Ti discs (10 mm diameter) were prepared as in Barkarmo et al. [36] by machining from commercially pure titanium grade 4 according to ISO 5832‐2/ASTM F67 (Zapp Medical Alloys GmbH, Schwerte, Germany*)*. Rough Ti samples were prepared by replicating the commonly used SLA (sand blasted, large-grit, acid etched) surface for dental implants, by sand blasting and acid etching the machined Ti discs as outlined in Vyas et al. [37] The Thermanox™ coverslips were used either in their original condition or were sandblasted to increase the surface roughness with 250 μm corundum particles (Korox, BEGO) (SANDIMAT, Local exhaust ventilation, Allianz Engineering Inspection Service Ltd, Italy). These were then cleaned in distilled water in an ultrasonic bath to dislodge any remnant particles from the surface and autoclaved at 121°C (1 Tor pressure, 15 min) to sterilise.

The average surface roughness of each sample was measured using a surface profilometer (Talysurf Series 2 inductive gauge profilometer, Taylor-Hobson, UK) as outlined in Wang et al. [38] Briefly, clean samples without any biofilm growth were measured using a 2 μm diamond tip and the roughness average (Ra) was calculated (μltra version 5.1.14, Taylor-Hobson, UK).

### Biofilm growth

The Gram-positive bacterium *Streptococcus sanguinis* (ATCC 10556) was used in the current study to form a simplistic early biofilm model for understanding the cavitation phenomena. Briefly, the stock microorganisms were recovered from porous storage beads (Pro-Lab Diagnostics, UK) maintained at −80°C and initially grown on Tryptone Soya Agar (Oxoid, UK) media at 37 ˚C with 5% CO_2_ for 3 days. 2–3 single colonies were used to inoculate 10 ml of Brain Heart Infusion (BHI) broth (Oxoid, U.K.) supplemented with 1% sucrose (Fluka Analytical, UK), which was incubated at 37 ˚C, shaking at 88 rpm overnight until it reached approximately 10^9^ colony forming units/ml. This primary culture was serially diluted to 10^3^ cfu/ml in BHI broth.

Artificial saliva was added to the coverslips or Ti discs to promote biofilm formation, this was prepared according to the method described by Pratten et al. [39], with the following chemicals from Sigma, UK (unless stated otherwise) added sequentially to RO (reverse osmosis) water: - 0.35g/L sodium chloride (NaCl), 0.2g/L potassium chloride (KCL), 0.2g/L calcium chloride (CaCl2), 2g/L yeast extract, 1g/L lab lemco powder, 2.5g/L hog gastric mucin and 5g/L proteose peptone. Reagents were mixed on a magnetic stir plate (Fisher scientific, Loughborough, UK) at ambient temperature for 1 hour. After autoclaving 1.25 mL of 40% sterile filtered urea (0.22 μm filter) was added to 1 L of the prepared artificial saliva. The prepared media was wrapped with aluminium foil to exclude light and prevent protein degradation [40] before being stored at 4 ±1°C. One ml of the artificial saliva was pipetted into each well of a 24-well plate into which a sterile Thermanox™ coverslip/Ti disc (13 mm, Nunc, ThermoFisher Scientific) had been placed and was removed after 15 minutes, to condition the samples. One side of the coverslips was bent upwards using sterile forceps to create a lip so the samples could be removed from the well with minimal biofilm disruption.

One ml of the diluted *S*. *sanguinis* culture and 1 ml of fresh BHI medium was added to each well of the 24-well plates. The 24-well plates were then incubated at 37 ˚C, 88 rpm for 24h to allow biofilm formation. The broth was replaced with 2 ml fresh BHI medium every 24 h. The Thermanox™ coverslips and Ti discs were removed from the 24 well plates after a total of 7 days of incubation and then fixed in 0.1 M sodium cacodylate buffer and 2.5% glutaraldehyde (25% EM grade, Agar Scientific, Essex, UK). They were then stained with Crystal Violet stain (0.5%, Pro-Lab Diagnostics, UK), pH 7.3, for 5 minutes and gently washed in Phosphate Buffered Saline (PBS) (Sigma-Aldrich, USA). Samples were stored in PBS until high speed imaging to prevent dehydration. The biofilm thickness was on the order of tens of microns, less than 100 μm. In previous studies *in vivo* and *in vitro* dental biofilms, which were 1–4 days old, were between 20–50 μm thick [41, 42], therefore the thickness of the biofilms in our study was similar to dental implant biofilms.

### High speed imaging

**Fig 2:**
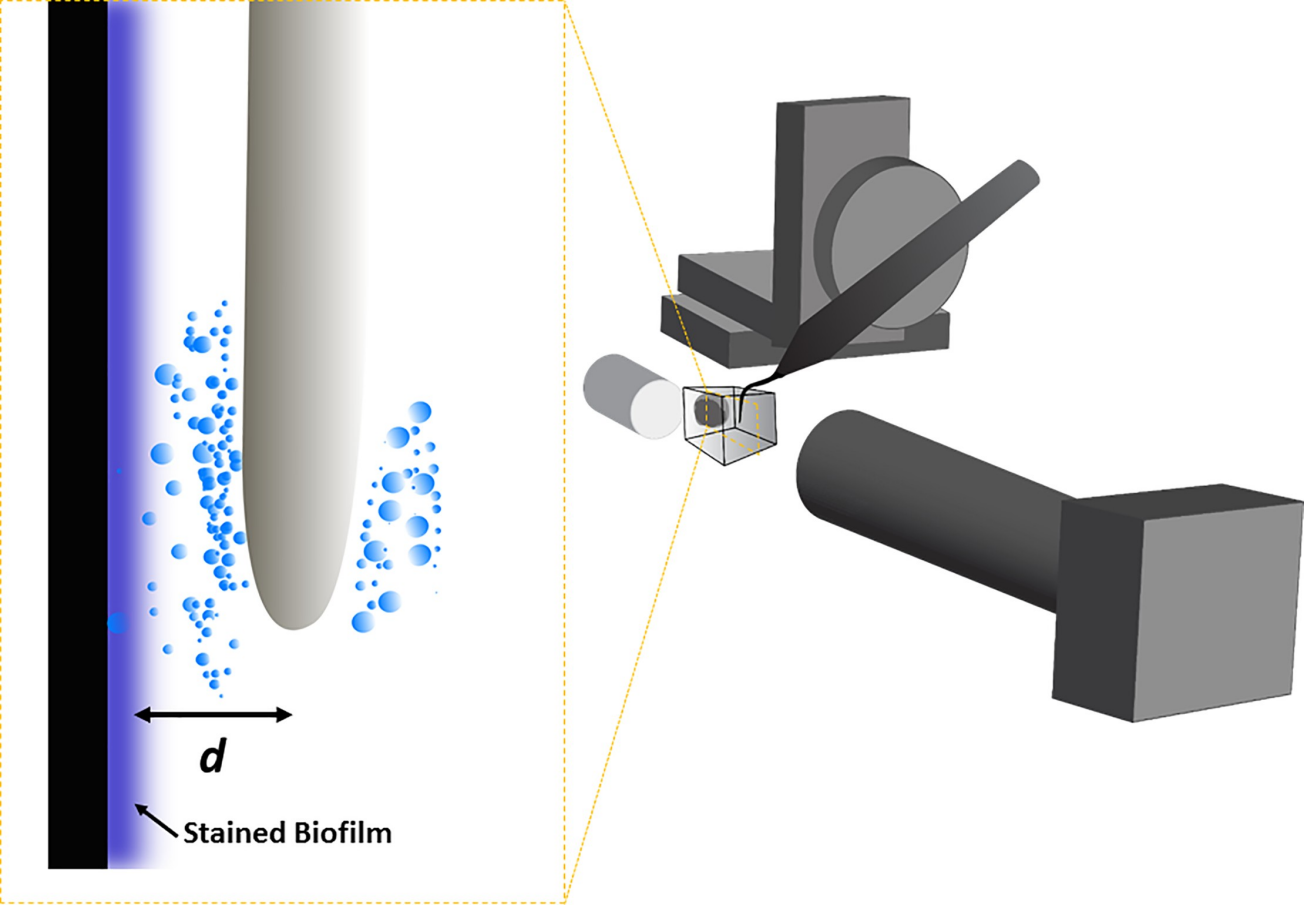
Schematic of the experimental setup for high speed imaging. Inset: schematic of the ultrasonic scaler tip being held close to the biofilm surface for experiments. The arrow indicates the standoff distance *d* of the scaler tip from the biofilm that was varied in this study from 0.5 mm to 2 mm.

High speed imaging was used to image biofilm removal from the different surfaces via cavitation (Fig 2). The substrate with biofilm was fixed vertically in a custom-made glass water tank with dimensions 2.7 cm x 2.7 cm x 2.7 cm. The tank was filled with 15 ml RO water. A P5 Newtron XS dental ultrasonic scaler (Satelec, Acteon, France) was used in conjunction with Tip 10P to generate the cavitation. The tip was immersed in the glass water tank and its position was fixed by attaching it to a XYZ translation stage (PT3, Thorlabs Inc, NJ, USA) and a high-precision rotation mount (PRO1/M, Thorlabs Inc, NJ, USA). The axial rotation of the scaler tip was also maintained during each experiment. The sample was illuminated using an LED cold light source (Hayashi HDF7010, Japan) in bright field mode for the transparent coverslips or in reflectance mode for the opaque Ti samples. The biofilm removal was imaged using a high speed camera (Fastcam mini AX200, Photron, Japan). A long distance microscope zoom lens was attached to the camera (12x zoom lens system, Navitar, USA) with a 2x adapter, giving a working distance of 32 mm. The scaler tip was operated at medium power (power 10) for 2s, at either 0.5 mm, 1 mm or 2 mm away from the biofilm. The standoff distance was measured from the attachment surface. Five samples were imaged for each test condition. High speed imaging was done at 500 frames per second (fps), at a magnification of x2 giving a resolution of 5 μm/pixel. The shutter speeds were 1/1000 s for the Ti samples, 1/20,000 s for the rough Thermanox™ coverslips or 1/300,000 for the smooth Thermanox™ coverslips. Although biofilm was removed in a circular pattern on both sides of the tip, high speed images were taken of only one part of the surface. Imaging was done in this way to keep the ultrasonic scaler tip at the side of the image so it did not obstruct the field of view during image analysis.

### Scanning electron microscopy

Scanning electron microscopy (SEM) was done to image the implants at high magnification after the biofilm disruption experiments using an EVO MA-10 (Zeiss, Germany). Images were taken at x1500 magnification. Samples were dehydrated using serial ethanol gradient immersions followed by hexamethyldisilizane evaporation and then gold sputter-coated (Emitech K550X, Kent, UK) for SEM as previously described [43]. Images were taken of undisturbed areas of biofilm and of the areas disrupted by cavitation. Secondary electron images were captured with an accelerating voltage of 10 kV and a working distance of 9.5 mm.

### Image analysis

**Fig 3:**
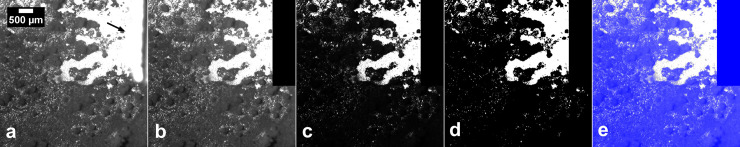
Example of a high speed image still, showing the image analysis steps performed to calculate the area cleaned (white). The image is taken from a video where the standoff distance between the ultrasonic scaler tip and the biofilm was 2 mm. The ultrasonic scaler used was operated at 29 kHz, with a tip vibration amplitude of 57 μm at the free end. The biofilm in this image was grown on rough (sand blasted and acid etched) titanium discs, Ra = 2 μm: (a) raw high speed image still showing the biofilm on the rough titanium surface, the black arrow shows the position of the scaler tip (b) image after the ultrasonic scaler tip was removed, (c) Image after gamma filter applied, (d) segmented image using automatic thresholding, and (e) overlay of the segmented image in blue on the raw image demonstrating that the segmentation is accurate.

Image analysis was used to gain quantitative measurements from the high speed image data. The total area cleaned was calculated by measuring the biofilm in each image before and after applying the cavitation, and the area of biofilm removed at each time point was also calculated (Fig 3). Image analysis was done using Fiji (ImageJ, U.S. National Institutes of Health, Bethesda, Maryland, USA) [44]. The biofilm area cleaned was calculated every 0.1 s for each test condition (*n* = 5). First, the ultrasonic scaler tip was removed from the images by replacing the pixels with a black rectangle of a fixed size to ensure the tip did not appear in the area calculations. Thresholding was used to segment the images to calculate the cleaned area. For the smooth Thermanox™ surfaces, the default automatic thresholding method was used. For the rough Thermanox™ surfaces, the background was first subtracted using a rolling ball radius of 3000 to ensure even illumination and the minimum automatic threshold was applied. For the rough Ti surfaces, a gamma filter was applied with a value of 2.74 to increase contrast between the biofilm and the background before applying the minimum threshold. These automatic thresholding methods were chosen after testing all automatic thresholds in ImageJ and finding the optimum method for each surface. The histogram was then calculated on the image stack to calculate the number of white pixels in each image which corresponded to the cleaned area. This was converted to a percentage area cleaned and averaged over 5 repeats. Due to uneven reflections on the surface of the smooth titanium surfaces it was not possible to segment the image by applying a threshold to calculate the area removed at each time point, therefore manual segmentation was used for this particular surface, on images taken before biofilm removal and 2s after biofilm removal. Data analysis and graphing was done using Sigmaplot and statistical significance was tested using the Kruskal-Wallis One Way Analysis of Variance on Ranks.

## Results

### Total biofilm removal

**Fig 4:**
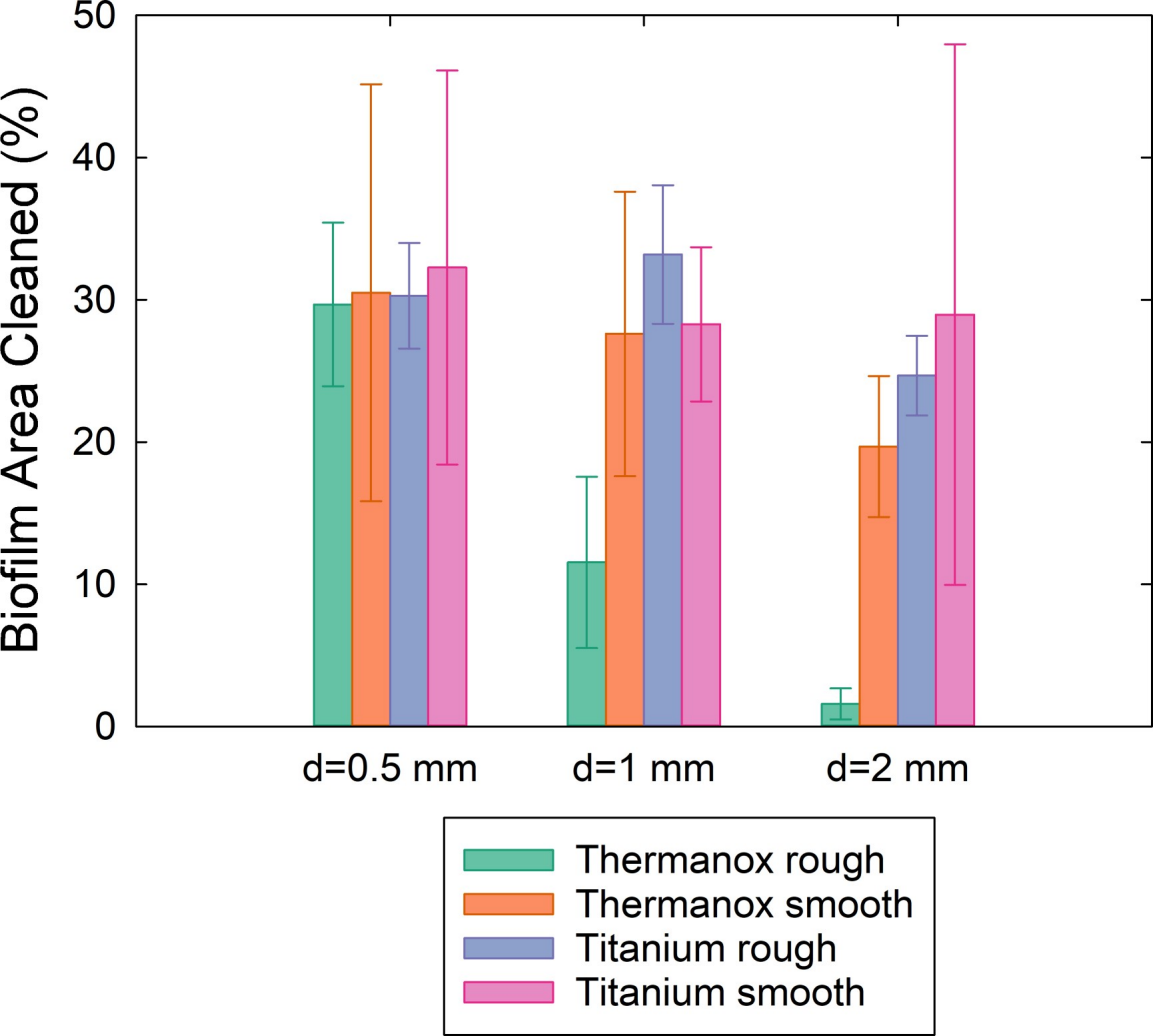
Total biofilm removal for the surfaces tested, removed using cavitation from an ultrasonic scaler tip at various standoff distances *d*, calculated using image analysis from high speed images. The area cleaned was calculated relative to the initial amount of biofilm in each image (n = 5).

The roughness (*Ra*) of the rough titanium surface was approximately 2 μm and the rough Thermanox™ surface had a *Ra* of approximately 3 μm (Table 1). The rough Thermanox™ surface was cleaned considerably less than the rough Ti at 1mm and 2mm (Fig 4, S1 Video). The rough Ti surface had similar amounts of biofilm removal to Ti smooth and Thermanox™ smooth surfaces at all standoff distances (Fig 4).

**Table 1:** Representative surface roughness (*Ra*) of the samples tested in this study, measured using surface profilometry.

	Thermanox™ rough	Thermanox™ smooth	Titanium rough	Titanium smooth
*Ra* (μm)	3.55±0.11	0.02±0.001	2.15±0.13	0.18±0.01

For nearly all surfaces the most biofilm removal occurred when the scaler tip was held 0.5 mm away from the biofilm, with similar amounts of biofilm removal (30–35%), irrespective of the surface roughness (Fig 4). There was one exception for the rough Ti surface, where slightly more cleaning occurred at 1 mm compared to 0.5 mm. In general biofilm removal decreased by 5–10% at 2 mm compared to 0.5 mm, but for the Thermanox™ coverslips, a higher surface roughness resulted in less biofilm disruption, decreasing from approximately 30% at 0.5 mm to approximately 10% at 1 mm and less than 5% at 2 mm.

### Biofilm removal over time

**Fig 5:**
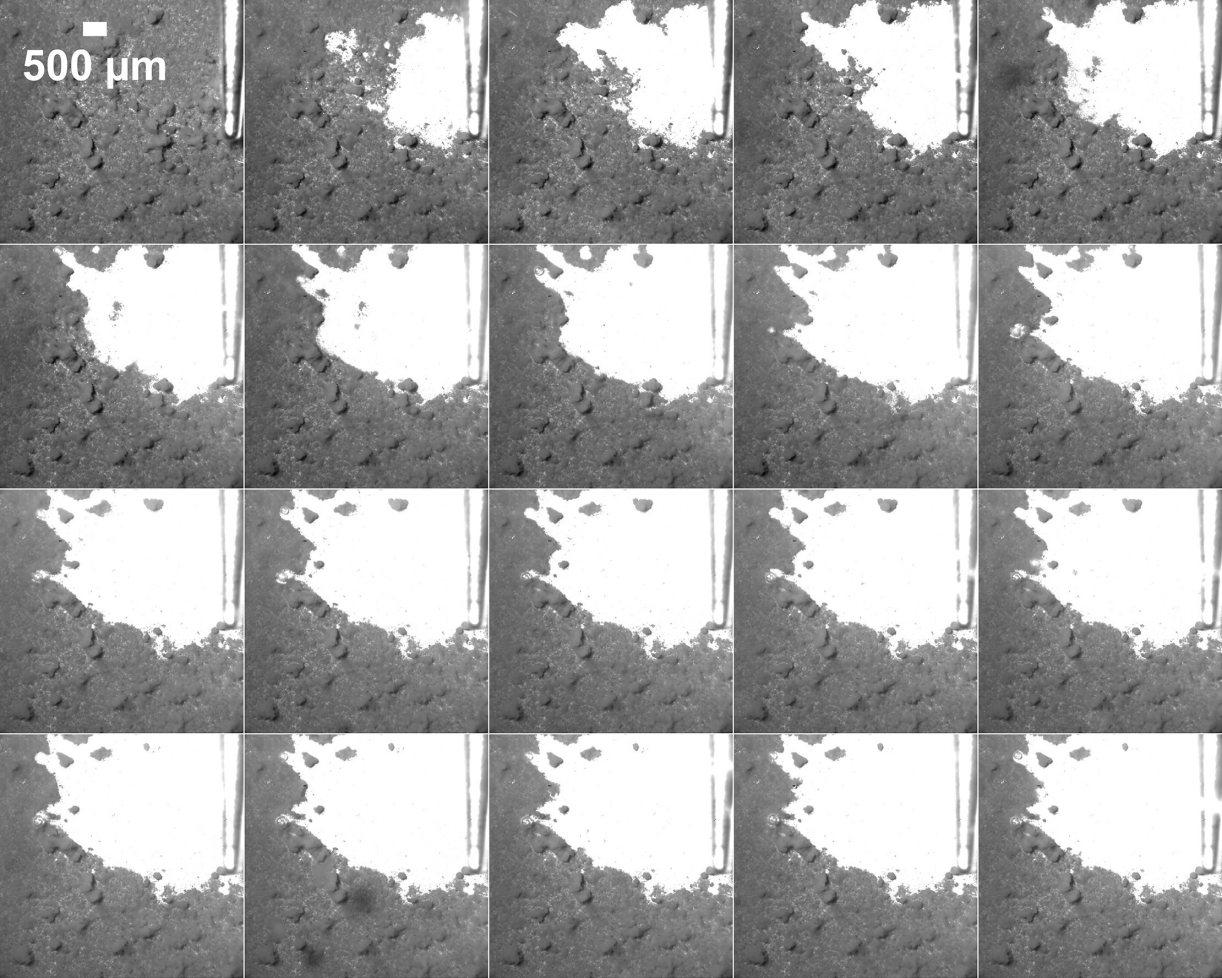
High speed video stills showing biofilm being removed from a rough Ti surface using ultrasonic cavitation at a standoff distance of 0.5 mm. Time between frames is 0.1 s (total 2 s). The cleaned area is white and the dark area is where biofilm is still attached to the surface. See S1 Video for high speed videos of all surfaces at all standoff distances.

**Fig 6:**
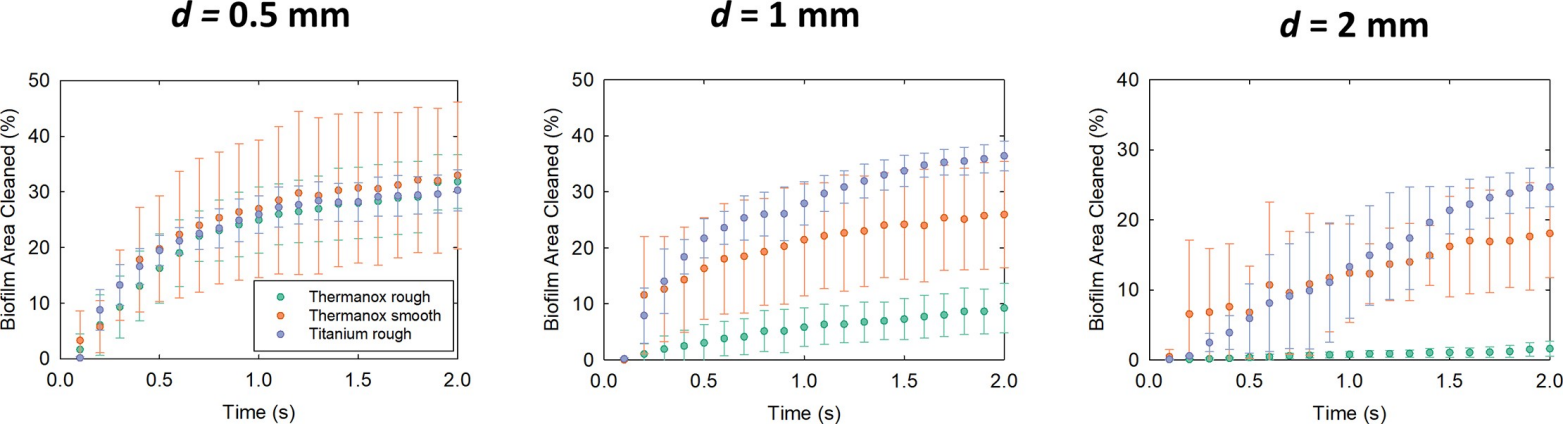
Percentage biofilm area cleaned over time for three of the surfaces tested using cavitation at three different standoff distances, calculated using image analysis. Green: Thermanox™ rough, Orange: Thermanox™ smooth, Purple: Titanium rough.

The majority of the biofilm removal occurred within the first 0.5s (Figs 5 and 6). After 0.5s the rate of cleaning reduced and almost stopped by the last image frame at 2s (Fig 6). At 0.5 mm, the removal rate over time and the total area removed were similar for all of the surfaces analysed, as the values of area cleaned at each time point follow a similar pattern (Fig 6A). At 1 mm and 2 mm the fastest rate of removal was from rough Ti surfaces. The slowest removal was from the sand blasted Thermanox™ surfaces.

### Qualitative observations of biofilm removal

**Fig 7:**
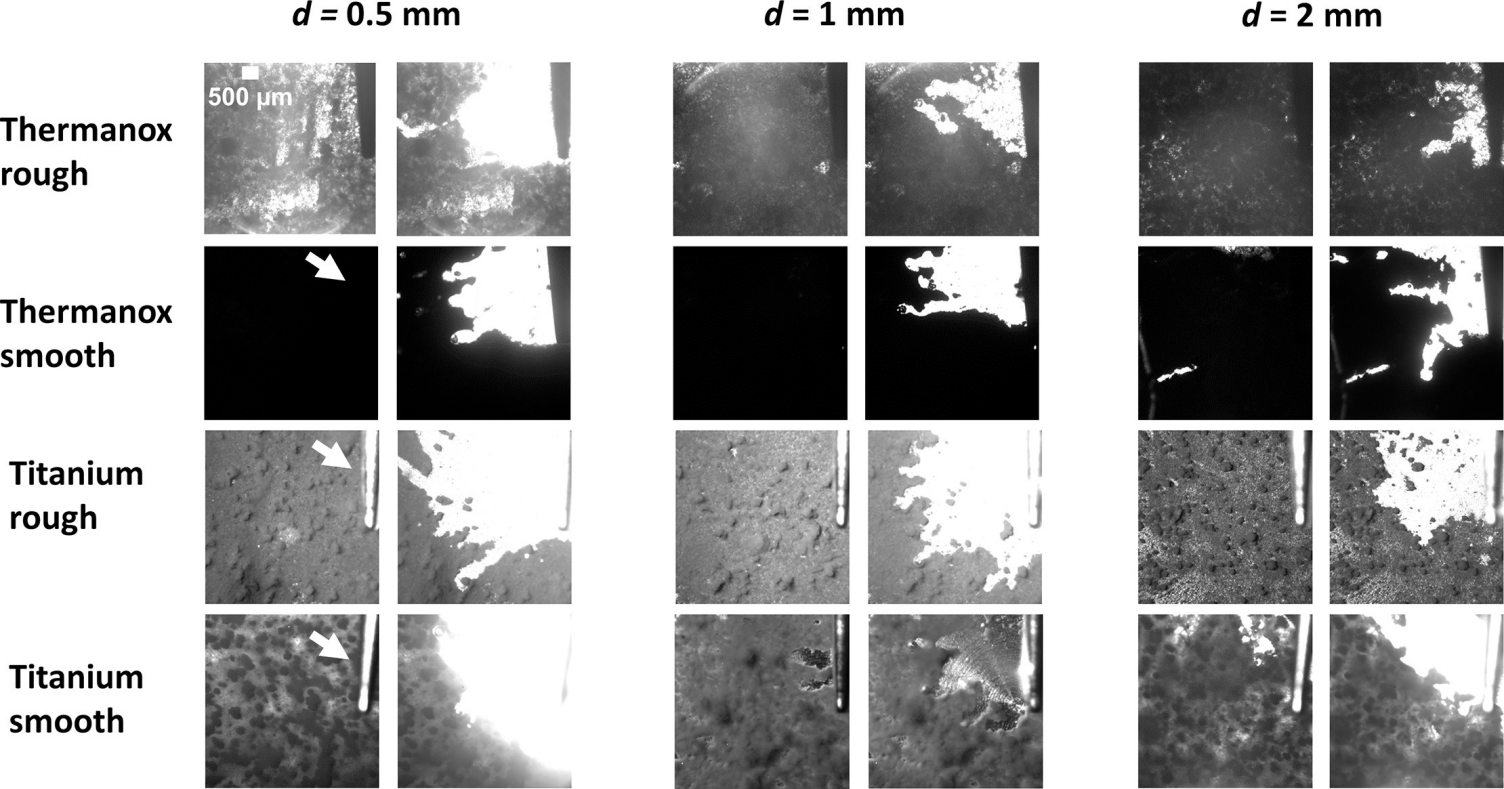
High speed image stills before and after cavitation cleaning for 2 s for various biofilm surfaces and standoff distances of the ultrasonic scaler tip. The white arrow shows the position of the scaler tip in the images. The white area is the cleaned surface and the grey/black area is the biofilm still attached to the surface. See S1 Video for high speed videos of all surfaces at all standoff distances.

The biofilm was removed in a radial pattern perpendicular to the tip, and some channels of cleaned biofilm extended away from the main area cleaned (Fig 7, S1 Video). This pattern of biofilm removal was the same for all of the surfaces tested. The images show that as the standoff distance between the tip and the biofilm increased, the radial area cleaned was smaller. Biofilm removal started in the vicinity closest to the tip and then continued radially outward from the tip, but only in areas perpendicular to the tip and not below it.

**Fig 8:**
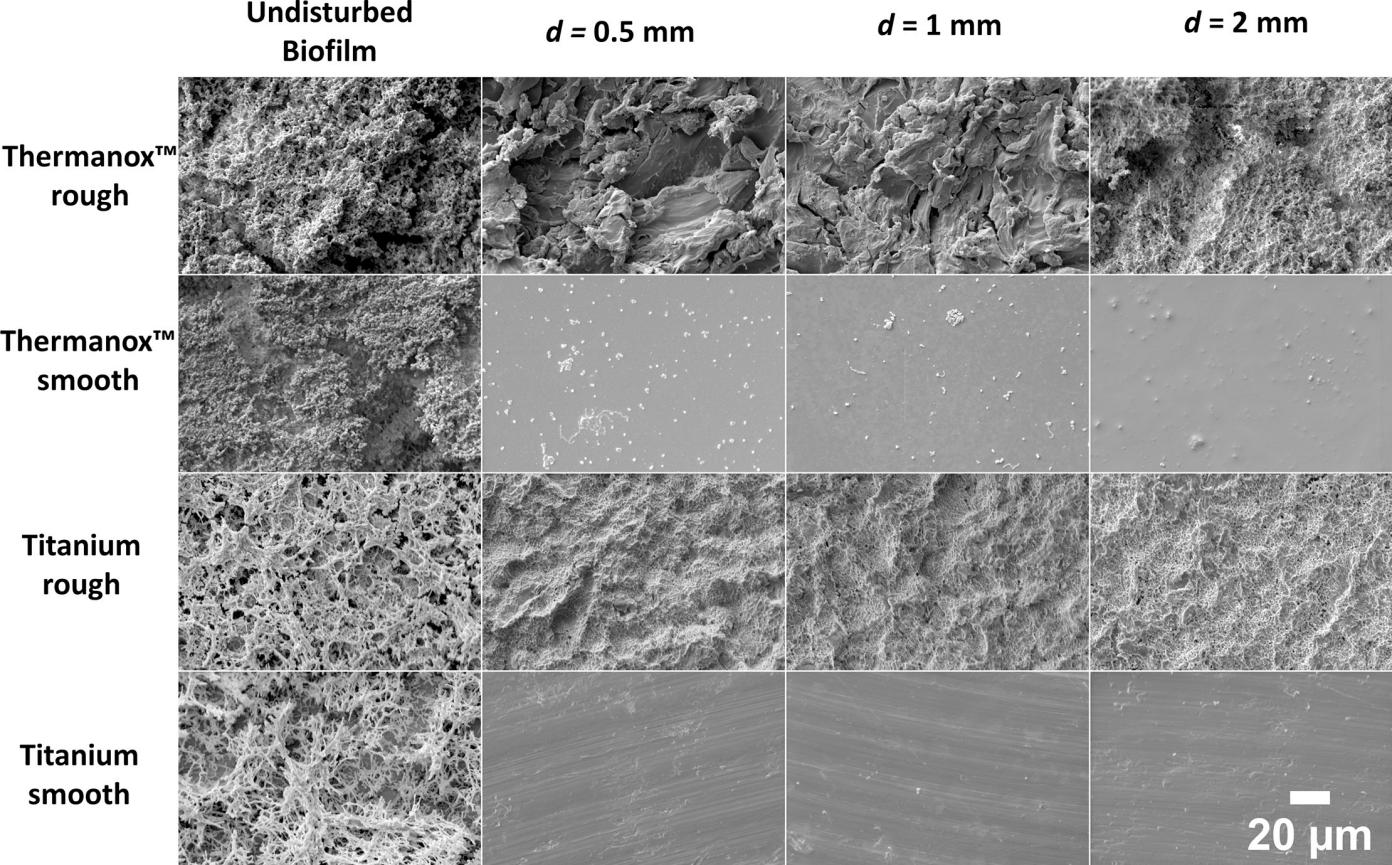
Scanning electron microscopy images of the different surfaces after removal with cavitation from the ultrasonic scaler tip at different standoff distances *d*. The ultrasonic scaler was operated at 29 kHz, with a tip vibration amplitude of 57 μm at the free end. Biofilm remained on the Thermanox™ rough surfaces at all distances, and individual bacteria remained on the Thermanox™ smooth surfaces at all standoff distances tested. Individual bacteria and thin biofilms remained on the rough titanium surfaces. The sand blasted and acid etched surface can be seen in the SEM images, and machining lines are visible on the smooth machined titanium surface.

SEM showed a thick layer of undisturbed biofilm on all of the surfaces where the cavitation did not cause disruption (Fig 8). In areas of biofilm disruption, no bacteria were visible on the smooth Ti surface after cavitation application at 0.5 mm, 1 mm and 2 mm. Some individual bacteria were seen on the Thermanox™ smooth surface in the disrupted area, at all 3 test standoff distances. On the sandblasted Thermanox™ surfaces, biofilm removal was not complete at any distance, but the most biofilm remained after cavitation at 2 mm, with thick multi-layer biofilms still present. There were small clusters of biofilm remaining in many areas on the disrupted surface at 0.5 mm and 1 mm, but these were thinner than the undisturbed biofilm (approximately 2–3 bacterial layers, determined by SEM). On the Ti rough surface, no bacteria or biofilm remnants were visible after removal at 0.5 mm or 1mm, but small biofilm clusters did remain at 2 mm.

## Discussion

This work shows that the cavitation produced in the water around the scaling tip produces forces that are able to remove biofilm from a surface. This removal is dependent upon how close the tip is in relation to the biofilm. The cavitation is most intense close to the tip and its intensity rapidly decreases with distance from the tip. Cavitation clouds occur around ultrasonic scalers tips, where bubbles chaotically grow and collapse in groups. The high speed images show chaotic oscillating bubbles on the surface cleaning the biofilm (S1 Video), therefore we speculate that the majority of biofilm disruption occurs through inertial cavitation. Previous experiments have shown they reach approximately 0.5 mm away from the tip and are approximately 2 mm wide [21]. Individual cavitating microbubbles have diameters of approximately 100 μm. Therefore in this study the cavitation clouds from the tip are likely to have reached the surface at a standoff distance of 0.5 mm, whereas at 1 mm and 2 mm the cleaning is likely to have only occurred from individual cavitating bubbles present on the biofilm surface. As more biofilm was removed at 0.5 mm, this suggests that the combined effect of cavitation clouds and individual bubbles on the surface is more effective. This would explain why at 0.5 mm the rate of biofilm removal was similar, even on the rough surfaces, on which the bacteria are expected to have a larger attachment force [45].

Previous studies have found that increased surface roughness leads to more biofilm accumulation [46]. Single species *S*. *sanguinis* biofilms have also been investigated and similar results were found, with more biofilm attaching to rough orthodontic composite materials and sand blasted and acid etched Ti surfaces compared to polished Ti surfaces [45, 47]. There are more possible points of bacterial adhesion on rough surfaces [45], which is thought to result in increased adhesion forces of bacteria to rough surfaces [45]. Previous studies found that the surface roughness of blasted and etched surfaces for dental implants was approximately 2 μm, similar to what was found in the current study [48]. The results in this study showed that the amount of biofilm removal on the rough Ti surfaces with a surface roughness of 2 μm or smaller was similar to that on the smooth surfaces, at all distances. This suggests that a surface roughness of 2 μm still enables effective removal with ultrasonic cavitation. However, a surface roughness of 3.5 μm significantly reduced the biofilm removal when the standoff distance was 1 mm or 2 mm (p<0.05). SEM images taken after the disruption showed that biofilm was still present behind larger sandblasted structures on the surfaces, which did not exist on the Ti sand blasted and acid etched surface. These differences suggest that the shear forces generated by the cavitation from an ultrasonic scaler operating at medium power are large enough to cause biofilm disruption from surfaces with a Ra value of 2 μm, but if the roughness is greater than this then the bacteria are sheltered from the shear forces by the larger surface structures. A systematic review found that the optimal surface roughness for osseointegration was 1–2 μm, and this is a commonly used surface roughness for dental implants [48]. Therefore, the results of this study suggest that clinically cavitation would be just as effective on implant collars as well as on the rougher surfaces of the implant which are prepared for osseointegration if their roughness is up to 2 μm. However, on areas of larger surface roughness such as scratches on the implant surface, or on implant surfaces with different treatments such as titanium plasma sprayed or hydroxyapatite coated which have a roughness of 9–10 μm [48], more intense cavitation would be required. This can be achieved by minimising the distance between the scaler tip and the surface to allow cavitation clouds to make contact, as was observed in the current study showing biofilm removal at 0.5 mm on the sandblasted Thermanox™ coverslips, but not at 2mm (Figs 4 and 8). Alternatively increasing the vibration amplitude of the ultrasonic scaler tip is likely to increase the volume of cavitation occurring [21], and further investigation is required to optimise this.

Interestingly the SEM images showed some individual bacteria remaining on the smooth Thermanox™ coverslips after treatment at all distances (Fig 8), but not on the polished Ti surfaces, which have a higher surface roughness compared to the smooth Thermanox surfaces™ (Table 1).

The results in this study show that biofilm removal can occur with cavitation from an ultrasonic scaler tip operating for only 2s. During clinical use of an ultrasonic scaler, the tip is moved around the mouth constantly to clean areas using the physical vibration of the scaler tip. Previous studies applied cavitation from an ultrasonic scaler for 15-60s [37, 49]. In these studies, biofilm was effectively removed, however such timescales are not practical for clinical application of the method where rapid biofilm removal from a larger area is required. The current results show that cavitation from an ultrasonic scaler may be a clinically viable method, if optimised for use on surfaces of different roughness and at the correct distance.

One limitation of this study is that the biofilm had to be fixed before imaging as the high speed camera could not be operated inside the microbiology laboratory due to laboratory safety requirements. Although the biofilm was kept hydrated and only immersed in the fixative for 10 minutes, it may have slightly different structural properties to a vital biofilm. Therefore in further work the high speed camera could be operated in a category II microbiology lab, with the biofilm inside a laminar flow hood during disruption to minimise aerosol. This experimental setup would also enable correlative studies using fluorescence microscopy where live/dead staining can be done to understand the effect of cavitation on biofilm viability. Also, in this study a single species biofilm was used, which may have different attachment properties to dental biofilm which is composed of many hundreds of species of bacteria. *S*. *sanguinis* was used in the current study because it is a primary coloniser on oral surfaces [50], and the use of a single species ensured reproducible biofilms. The effectiveness of sonication for physical disruption has been imaged on other biofilm species in other studies, for example Rivas et al. imaged *Enterococcus faecalis* and *Pseudomonas aeruginosa* biofilms being removed with cavitation generated by a custom made vibrating ultrasonic surface [51]. Biofilm removal occurred in a similar manner, therefore we anticipate that cavitation from an ultrasonic scaler would also behave similarly on other bacterial species. Further work is required to image biofilm disruption using multispecies biofilms, and to compare removal from biofilm grown in a shaking incubator, a bioreactor or a flow cell to determine if the hydrodynamic stress affects its adherence. Song et al. highlighted the need for research to understand how material properties affect bacterial adhesion to surfaces [30]. In the current study we have developed a novel protocol of imaging and image analysis to quantify biofilm removal over time from high speed images. This novel method can be applied to future studies to investigate other methods of biofilm disruption, including biofilm rate of removal from different surfaces such as those with novel anti-biofilm topographic patterns, and also in studies investigating methods of physical biofilm disruption.

There may also be other factors regarding the biofilm properties which could affect their removal ability via cavitation. Although biofilm thickness increases with its age, we speculate that the surface roughness would have more effect on the effectiveness of sonication rather than biofilm age or thickness, as the surface roughness affects the attachment force of the first bacterial layer, but this requires further investigation. Dental biofilm also mineralises with age to become dental calculus, which is difficult to remove. Further work is required to investigate the effect of cavitation of dental calculus. A combination of cavitation with using the vibrating tip of the ultrasonic scaler in contact may be required.

Damage to the implant surface was not observed in SEM images in the current study and also in a previous study where the scaler tip was held for 60 s [37]. From this we speculate that cavitation from an ultrasonic scaler does not damage tooth and biomaterial surfaces in the mouth. In considering sonication for clinical use to remove oral biofilms, it is important to consider the effects that cavitation could have on soft tissue in the mouth. The cavitation is likely to contact the gums surrounding a tooth or dental implant. In current clinical practice the vibrating ultrasonic scaler tip contacts these soft tissues and can lead to some gingival bleeding. It is possible that with sonication some relatively mild gum damage may occur which would heal within a few days, similarly to what occurs after using an ultrasonic scaler in the conventional manner. This is because an elastic or soft boundary weakens or redirects the cavitation bubble jetting away from the soft surface, resulting in a smaller hydrodynamic load on the soft surface [52, 53]. We therefore speculate that cavitation would cause less damage than what is currently occurring clinically, since the metal tip would not be touching the surface. Further studies are required to understand the damage caused and how it can be minimised, such as by directing the cavitation towards the hard surface to be cleaned.

## Conclusions

In conclusion, high speed imaging has shown biofilm removal via cavitation generated by an ultrasonic scaler in real time on rough and smooth implant surfaces and at different standoff distances up to 2mm. We observed significant biofilm removal after operating an ultrasonic scaler tip for only 2s. Cavitation from an ultrasonic scaler is more effective when the tip is closer to the surface, and is equally effective on smooth and rough (SLA-like) titanium surfaces, demonstrating its potential as a novel method of dental implant debridement.

## Supporting information

S1 Video High speed video of biofilm removal. High speed videos taken at 500 frames per second showing biofilm removal from all of the surfaces tested using cavitation from an ultrasonic scaler, at different standoff distances. (a) rough (sandblasted) Thermanox™ surface (b) smooth Thermanox™ surface (c) rough (sand blasted and acid etched) titanium surface (d) smooth (machined) titanium surface.
(AVI)
